# Comparative Analysis of Microbial Community Structure and Function in the Gut of South China Tigers Under Different Dietary Treatments

**DOI:** 10.3390/vetsci13030307

**Published:** 2026-03-23

**Authors:** Qiguan Qiu, Tengfang Gong, Lin Du, Wenchao Li, Yuanpeng Hu, Dianshu Li, Caiwei Zhou, Wei Liu

**Affiliations:** 1 Changsha Ecological Zoo, Changsha 410100, China; qiuqiguan163@163.com (Q.Q.); gongtf@stu.hunau.edu.cn (T.G.); 2 Research Center for Parasites & Vectors, College of Veterinary Medicine, Hunan Agricultural University, Changsha 410128, China; dulinb20251148@stu.hunau.edu.cn (L.D.); huyuanpeng1027@163.com (Y.H.); 17670776096@163.com (D.L.); 2507336973@stu.hunau.edu.cn (C.Z.); 3 Changsha Customs Technology Center, Changsha 410004, China; liwenchao0725@163.com

**Keywords:** South China tigers, gut microbiota, metagenomic, dietary

## Abstract

The gut microbiota is a crucial component of a tiger’s health and plays a significant role in adapting to changes in food and the environment. Although extensive studies have been carried out on the gut microbiota of tigers, investigating the responses of gut microbial composition and function to preadaptation to wild predation patterns under captive conditions is particularly significant for South China tigers, given that it is the only tiger subspecies existing solely in captive settings at present. Here, we performed shotgun metagenomic sequencing for a comprehensive analysis of the gut microbiota of South China tigers assigned to two dietary groups (live prey group, LP group; frozen meat group, FM group), thereby generating abundant valuable data for this endangered subspecies. The results indicated that the core intestinal microbial composition was similar between the two dietary groups. Differential analysis revealed associations between dietary treatments and microbial abundance in the intestines of South China tigers. Functional gene analysis revealed that the LP group exhibited upregulation of genes and pathways related to antimicrobial resistance, bacterial infection-related disease, cell motility and proliferation, while the FM group displayed efficient energy metabolism. A total of 1251 antibiotic resistance genes (ARGs) were identified in the gut microbiome of South China tigers. The core resistome mainly included resistance to peptides, glycopeptides, tetracyclines, fluoroquinolones, and macrolides. In addition, the differences in ARGs between the LP group and FM group may be related to a broader range of animal tissues of live prey and the processing conditions of frozen meat. In summary, although feeding live prey did not change the core framework of the gut microbiota in South China tigers, it was associated with differences in microbial abundance, metabolic pathways, and antibiotic resistance gene profiles.

## Simple Summary

Compared with captive feeding, dietary changes in the wild environment can significantly alter the structure and function of tigers’ intestinal microbiota. This may have an impact on the health and adaptability of the host, as well as the success rate of reintroduction. Here, we performed shotgun metagenomic sequencing for a comprehensive analysis of the gut microbiota of South China tigers, assigning them to two dietary groups (LP group; FM group), thereby generating abundant, valuable data for this endangered subspecies. Therefore, we believe that this manuscript would provide scientific support for optimizing precision feeding schemes for South China tigers, reducing the risk of diseases and the transmission of drug-resistant bacteria, and also offer important references for improving the pre-reintroduction health assessment system and formulating conservation strategies for endangered felines.

## 1. Introduction

The South China tiger (*Panthera tigris amoyensis*) is a tiger subspecies endemic to China and is recognized as one of the world’s most critically endangered large carnivores [1]. Historically, the South China tiger was widely distributed across central and southern China, where it played a critical role in maintaining the dynamics of forest ecosystems. However, prolonged anthropogenic pressures, including habitat fragmentation and historical overexploitation, have led to its extinction in the wild. Currently, the global population comprises approximately 200 individuals, all of which are maintained under captive conditions [2,3,4]. In recent years, with the advancement of conservation efforts for endangered species, the breeding and reintroduction into the wild of the South China tiger have increasingly become major research priorities [5,6].

Over the past decades, extensive scientific and clinical research has established a well-recognized symbiotic relationship between hosts and their gut microbiota. Gut microbial communities play fundamental roles in maintaining host health, including food digestion and nutrient absorption, regulation of intestinal immunity and homeostasis, suppression of pathogenic microorganisms, detoxification processes, and the integrated regulation of host physiological functions [7,8]. Importantly, both the composition and functional potential of the gut microbiome are highly responsive to host diet and lifestyle [4,9]. Reintroduction of captive individuals into natural habitats is widely regarded as an effective strategy for restoring wild populations and maintaining genetic diversity in endangered species. However, captivity-associated shifts in diet and living environment can profoundly alter gut microbial structure and function in wildlife species, with potential consequences for host health, fitness, and reintroduction success [10]. In recent years, increasing attention has been paid to the gut microbiome of tiger subspecies, with comparative analyses revealing pronounced differences between individuals inhabiting wild and captive environments. These findings highlight the importance of gut microbiome integrity in conservation and rewilding programs [1,11,12,13,14]. However, unlike other tiger subspecies, the absence of extant wild South China tigers precludes direct assessment of how natural diets and environments shape their gut microbiome. This knowledge gap represents a major bottleneck for evaluating captive suitability and predicting post-reintroduction adaptability.

Compared with 16 S rRNA gene amplicon sequencing, metagenomic sequencing enables not only comprehensive taxonomic profiling of microbial communities but also indepth characterization of functional genes, including metabolic pathways, environmental adaptation mechanisms, and ARGs. Owing to these advantages, metagenomics has been widely applied to investigate microbial community structure, functional potential, gene activity, and microbe–environment interactions [15,16,17]. Given the lack of wild South China tiger populations and the limited baseline data available, the present study was designed primarily as an exploratory study rather than a hypothesis-testing study. Specifically, we aimed to characterize the gut microbiome composition, functional metabolic potential, and ARG profiles of captive South China tigers under two dietary regimes (live prey and frozen meat). Furthermore, we explored whether different feeding strategies were associated with variations in microbial community structure and functional gene composition. These results provide baseline information on the gut microbiome characteristics of captive South China tigers and may offer preliminary insights relevant to future studies on dietary management and conservation strategies for this critically endangered subspecies.

## 2. Materials and Methods

### 2.1. Study Area and Sampling

Ten captive South China Tigers were selected from Changsha Ecological Zoo in Hunan Province, China. These tigers were aged 11 years, with an equal ratio of males to females, similar health status, and no history of recent antibiotic use. They were divided into two groups of five tigers each, and each tiger was housed and fed individually. Each tiger was kept in an enclosure cage of approximately 20 m^2^, with an outdoor activity area of about 150 m^2^. The LP group was provided with unprocessed natural prey (such as live poultry, rabbits, sheep, and unfrozen beef and pork) to simulate the foraging pattern in the wild environment. The FM group was fed artificially cut, lightly processed frozen meat (such as cut beef, pork, and chicken), which had been stored frozen at −20 °C. The frozen meat was thawed to room temperature before feeding and cut into pieces of approximately 0.5–1 kg. Prior to formal sampling, a one-month acclimation period was conducted to allow the animals to adapt to the dietary treatments. In addition, feeding and enclosure cleaning were consistently performed by the same caretaker throughout the experimental period to minimize environmental variability. The research complied with the protocols established by the China Wildlife Conservation Association and the legal requirements of China.

From May 2025 to September 2025, fresh fecal samples were collected from the 10 South China Tigers, respectively. When the South China tigers were released outdoors, the door was closed, and researchers entered the enclosure to collect fresh fecal samples. Each tiger was sampled once every 21 days, for a total of six consecutive sampling events. During each sampling round, five fecal subsamples were collected from the same individual to reduce sampling bias and capture within-individual variation. In this study, each tiger was treated as the basic biological unit. During sampling, researchers wore sterile gloves, transferred the central part of each fecal sample into a 100 mL sterile centrifuge tube, and recorded the individual identity and collection time. All samples were immediately transported to the laboratory on dry ice after collection and stored at 80 °C for subsequent research.

### 2.2. DNA Extraction, Library Construction, and Metagenomic Sequencing

DNA extraction from fecal samples was performed using the BayBiopure Magnetic Soil DNA Kit (BayBio, Guangzhou, China). Following extraction, DNA concentration and purity were assessed, and the extracted DNA was checked on 1% agarose gel. Extracted DNA was fragmented to an average size of about 350 bp using Covar’s M220 (Gene Company Limited, Hong Kong, China) for paired-end library construction. Paired-end library was constructed using the NEXTFLEX^®^ Rapid DNASeq Kit (Bioo Scientific, Austin, TX, USA). Specifically, adapter ligation was first performed, followed by magnetic bead purification to remove adapter dimer fragments. Subsequently, PCR amplification was carried out to enrich the library templates, and the final libraries were obtained via magnetic bead recovery of the PCR products. Metagenomic sequencing was performed on the Illumina NovaSeq X plus (Illumina, San Diego, CA, USA) by Majorbio Biopharm Technology Co., Ltd. (Shanghai, China), according to the manufacturer’s instructions (www.lumina.com, accessed on 10 October 2025).

### 2.3. Sequence Quality Control and Genome Assembly

The data were analyzed on the free online platform of Majorbio Cloud Platform (www.majorbio.com). Briefly, the paired-end Illumina reads were trimmed of adaptors, and low-quality reads (length < 50 bp or with a quality value < 20) were removed by fastp (https://github.com/OpenGene/fastp, version 0.23.0). Reads were aligned to the South China Tiger genome (GWHBEIN00000000) by BWA (http://bio-bwa.sourceforge.net/, version 0.7.9a) and any hits associated with the reads and their mated reads were removed. Metagenomics data were assembled using MEGAHIT (https://github.com/voutcn/megahit, version 1.2.9), which makes use of succinct de Bruijn graphs. Contigs with a length ≥ 300 bp were selected as the final assembling result, and then the contigs were used for further gene prediction and annotation.

### 2.4. Gene Prediction and Construction of a Nonredundant Gene

Open reading frames (ORFs) were identified from each assembled contig using Prodigal (http://metagene.cb.k.utokyo.ac.jp/). ORFs with lengths of at least 100 bp were retained and subsequently translated into their corresponding amino acid sequences with EMBOSS (http://emboss.openbio.org/, V6.6.0), based on the NCBI translation table. A nonredundant gene catalog was then generated using CDHIT (http://www.bioinformatics.org/cdhit/, version 4.6.1) with sequence identity and coverage thresholds both set to 90%. To estimate gene abundance, high quality sequencing reads were mapped back to the nonredundant gene catalog using SOAPaligner (http://soap.genomics.org.cn/, version 2.21) with a minimum identity cutoff of 95%.

### 2.5. Species and Functional Annotation

Representative sequences from the nonredundant gene catalog were aligned against the NR database (version: NR20241007) using Diamond (https://github.com/bbuchfink/diamond, version 0.8.35) with an evalue cutoff of 1 × 10^−5^. Taxonomic annotations were assigned based on the corresponding taxonomy information associated with the NR database. Species-level abundances were subsequently calculated by summing the abundances of genes annotated to the same taxon. The COG (version: COG 2020) and KEGG (version: KEGG 20241007) annotations for the representative sequences were performed using Diamond (https://github.com/bbuchfink/diamond, version 0.8.35) against the COG database and KEGG database, respectively, with an evalue cutoff of 1 × 10^−5^. Carbohydrate-active enzymes (CAZy, version: CAZy v12) annotation was conducted using hmmscan (https://www.ebi.ac.uk/Tools/hmmer/search/hmmscan) against the CAZy database (http://www.cazy.org/) with an evalue cutoff of 1 × 10^−5^. In addition, antibiotic resistance annotation was conducted using Diamond (https://github.com/bbuchfink/diamond, version 0.8.35) against the CARD database (https://card.mcmaster.ca/home) with an evalue cutoff of 1 × 10^−5^.

## 3. Results

### 3.1. Sequencing Data and Gene Prediction

**Figure 1:**
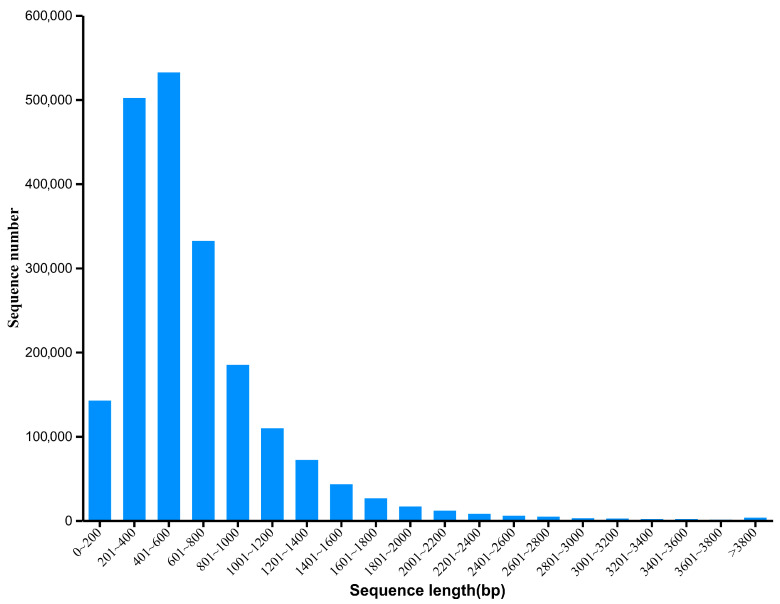
Length distribution of the nonredundant gene catalog.

The sequencing data of the South China tiger’s fecal samples in this study were acquired through the Illumina NovaSeq X plus (Illumina, San Diego, CA, USA) sequencing platform. 2,024,273.73 Mbp of raw reads were obtained from the metagenomic sequencing, and the average sequencing data volume was 6747.58 Mbp. The total and average data after quality control were 1,995,831.21 Mbp and 6652.77 Mbp, respectively. The effective data rate of quality control was 98.58%. A total of 38,056.40 bp scaffolds were assembled with an average length of 1666.82 bp, the maximum length of 523,081 bp, N50 of 2841 bp, and N90 of 646 bp. Specific data output statistics and quality control information are shown in Table S1. A total of 2,001,801 nonredundant genes were identified from all South China tiger fecal samples, with an average gene length of 650.05 bp, accounting for 4.02% of all predicted genes (Figure 1).

### 3.2. Species Annotation and Analysis

**Figure 2:**
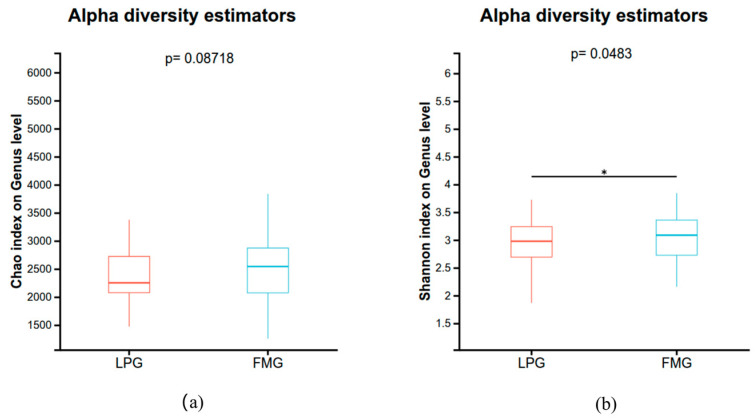
Alpha diversity of the gut microbiome in LP and FM groups. Chao (**a**) and Simpson (**b**) indices at the genus level. Significance levels: * 0.01 < *p* ≤ 0.05.

The analyses of bacterial alpha and beta diversity were conducted to investigate the similarities and differences in gut microbiome community structures among the two host species. The analyses using the Chao index suggested that gut microbial richness had no significant differences between the LP and FM groups (Wilcoxon test, *p* > 0.05; Figure 2a). However, Shannon analysis showed significant differences (Wilcoxon test, *p* < 0.05; Figure 2b), which were higher in the FM group than in the LP group.

**Figure 3:**
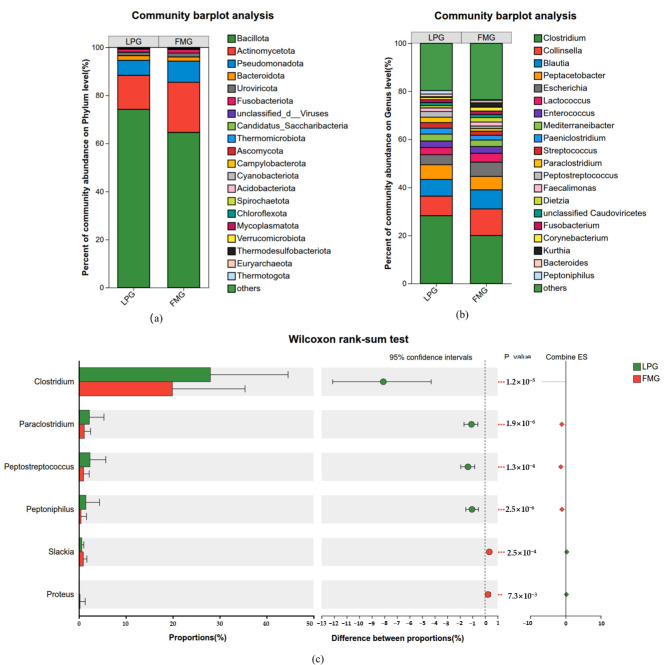
Overview of gut microbial communities in LP and FM groups. (**a**) The relative abundance of the top 20 phyla detected in fecal samples of South China tigers. (**b**) The relative abundance of the top 20 genera detected in fecal samples of South China tigers. (**c**) Significant differences in microbial abundance at the genus level between the LP and FM groups, based on the Wilcoxon ranksum test. Significance levels: ** 0.001 < *p* ≤ 0.01, *** *p* ≤ 0.001.

A total of 252 phyla, 482 classes, 902 orders, 1749 families, and 5966 genera were identified from fecal samples of South China tigers. At the phylum level, the microbial community comprised 168 bacterial phyla, 15 viral phyla, 41 eukaryotic phyla, and 28 archaeal phyla. In addition, six phyla were exclusively detected in the LP group, whereas 19 phyla were unique to the FM group. The relative abundances of the top 20 phyla are shown in Figure 3a, with the remaining taxa grouped as “Others”. At the phylum level, the fecal microbiome of the LP group was dominated by *Bacillota* (74.18%), followed by *Actinomycetota* (14.16%), *Pseudomonadota* (6.24%), *Bacteroidota* (1.94%), and *Fusobacteriota* (1.25%), while other phyla each accounted for less than 1% of the total relative abundance. In comparison, the FM group exhibited a lower relative abundance of *Bacillota* (64.58%), but higher abundances of *Actinomycetota* (20.85%) and *Pseudomonadota* (8.82%).

A total of 5966 genera were identified from fecal samples of South China tigers. At the genus level, the microbial community comprised 3970 bacterial genera, 717 viral genera, 1071 eukaryotic genera, and 208 archaeal genera. In addition to the commonly shared genera, both the LP and FM groups harbored a number of unique genera, with 172 genera exclusively detected in the LP group and 902 genera unique to the FM group. The top 20 genera with the highest relative abundances are shown in Figure 3b. Among the shared genera, the top 10 taxa were largely similar between the LP and FM groups’ fecal microbiomes and were primarily composed of *Clostridium*, *Collinsella*, *Blautia*, *Peptacetobacter*, *Escherichia*, *Lactococcus*, *Enterococcus*, *Mediterraneibacter*, and *Paeniclostridium*. The relative abundances of *Clostridium* in the LP and FM groups were 28.23% and 20.01%, respectively, indicating that this genus was dominant in both groups. The relative abundance of *Collinsella* was comparatively lower (8.11% in the LP group and 11.03% in the FM group). The remaining dominant genera, including *Blautia*, *Peptacetobacter*, *Escherichia*, *Lactococcus*, *Enterococcus*, *Mediterraneibacter*, and *Paeniclostridium*, exhibited relative abundances ranging from 1.95% to 7.95% across the two groups. A genus-level differential abundance analysis was conducted between the LP and FM groups. The results showed that *Clostridium*, *Paraclostridium*, *Peptostreptococcus*, and *Peptoniphilus* showed significantly higher abundances in the LP group than in the FM group, while *Slackia* and *Proteus* showed significantly lower abundances (Figure 3c).

**Figure 4:**
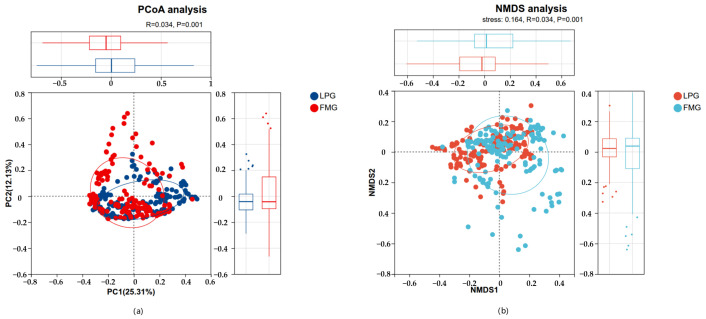
PCoA and NMDS analyses of microbial community composition at the genus level. (**a**) PCoA of microbial community composition in the LP and FM groups based on Bray–Curtis distances. (**b**) NMDS of microbial community composition in the LP and FM groups based on Bray–Curtis distances. The significance of differences in microbial community composition between groups was assessed using ANOSIM.

The fecal microbial communities of the LP and FM groups exhibited distinct community compositions, with clear differences between the two groups observed in both PCoA and NMDS analyses (*p* < 0.05; Figure 4). These differences are likely associated with the contrasting feeding regimes, suggesting that dietary variation may influence the gut microbiota of South China tigers.

### 3.3. Functional Annotation and Analysis

2,001,801 genes identified from the collected fecal samples were analyzed for functional characterization. Out of the 2,001,801 genes, 1,196,003 (59.75%) genes were annotated using the COG database, 49,167 (2.46%) in the CAZy database, and 1,116,673 (55.78%) in the KEGG database.

**Figure 5:**
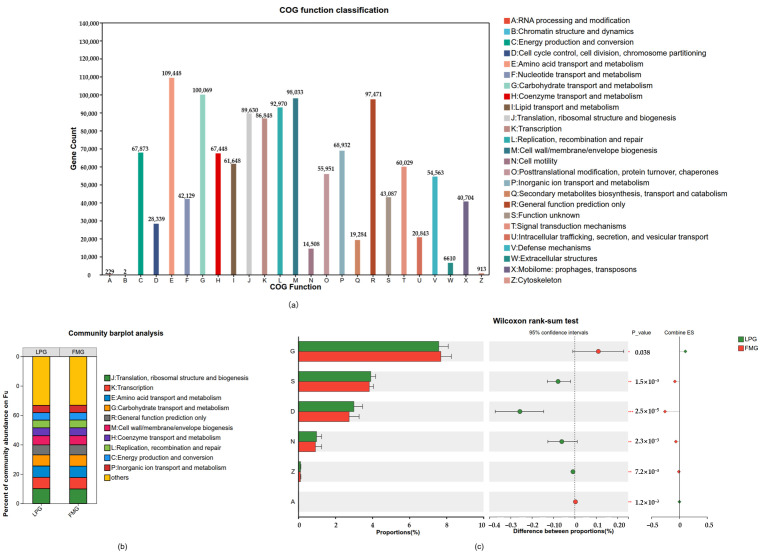
COG functional analysis of gut microbiota in LP and FM groups. (**a**) COG functional classification of annotated unigenes. (**b**) Relative abundance of COG functional categories in LP and FM groups (**c**) Differential COG functional categories between LP and FM groups identified by the Wilcoxon ranksum test. Significance levels: * 0.01 < *p* ≤ 0.05, ** 0.001 < *p* ≤ 0.01, *** *p* ≤ 0.001.

As per the functional analysis of genes using COG, except for the unknown functions category, 25 functional categories were enriched, and a majority of the genes were enriched in functional categories, such as amino acid transport and metabolism, carbohydrate transport and metabolism, cell wall/membrane/envelope biogenesis, and general function prediction only. Few genes were enriched in the RNA processing and modification, chromatin structure and dynamics, and cytoskeleton (each ≤0.01%) categories. The abundance proportions of LP and FM groups in each functional category are almost the same (Figure 5b). The abundance differences in various functional categories between the LP and FM groups were compared based on the Wilcoxon ranksum test. The results indicated that the LP group had significantly higher abundance than the FM group in the following categories: cell cycle control, cell division, chromosome partitioning, cell motility, and cytoskeleton. In contrast, the LP group showed significantly lower abundances than the FM group in the categories of Carbohydrate transport and metabolism, as well as RNA processing and modification (Figure 5c).

**Figure 6:**
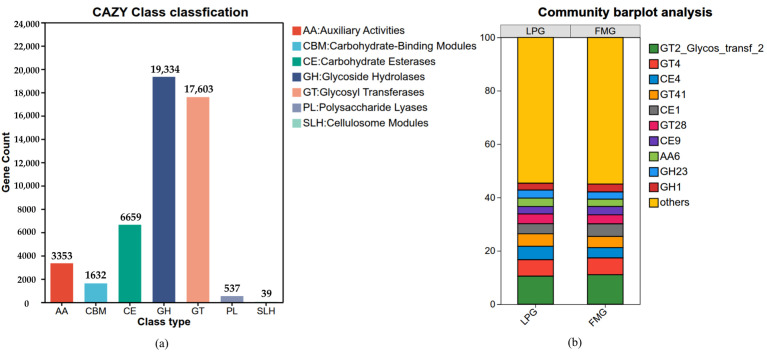
CAZy functional analysis of gut microbiota in LP and FM groups. (**a**) CAZy class classification of annotated unigenes. (**b**) The relative abundance of CAZy families.

In addition, we examined the changes associated with the altered CAZy gene abundance. In the CAZy analysis, the abundant enzymes in descending order of abundance were glycoside hydrolases (GHs), glycosyltransferases (GTs), carbohydrate esterases (CEs), auxiliary activities (AAs), carbohydrate-binding modules (CBMs), polysaccharide lyases (PLs), and cellulosome modules (SLHs) (Figure 6a). At the family level of CAZy classification, a total of 560 CAZy families were identified based on metagenomic analysis (Table S2). The relative abundance profiles of CAZy were nearly identical between the LP and the FM groups (Figure 6b). Among these, the GT2, GT4, CE4, GT41, and CE1 families were identified as the most abundant CAZy families across the collected fecal samples. Significant differences in the abundance of 238 microbial enzymes were detected between the LP and FM groups based on the Wilcoxon ranksum test. Among these, 75 enzymes showed significantly higher abundance in the LP group compared with the FM group, whereas 163 enzymes were significantly decreased (Table S3).

**Figure 7:**
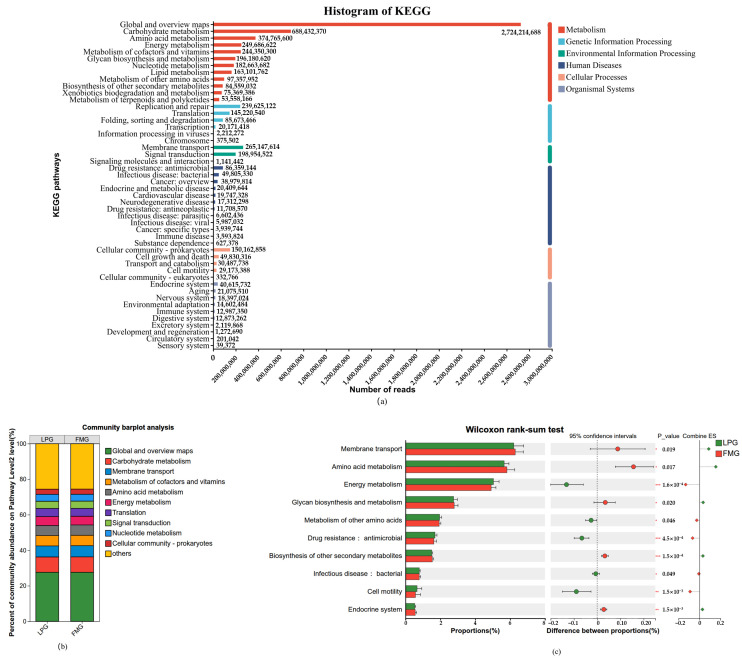
KEGG functional analysis of gut microbiota in LP and FM groups. (**a**) The number of functional annotations of genes. (**b**) The relative abundance of KEGG categories. (**c**) Differential KEGG functional categories between LP and FM groups identified by the Wilcoxon ranksum test. Significance levels: * 0.01 < *p* ≤ 0.05, ** 0.001 < *p* ≤ 0.01, *** *p* ≤ 0.001.

Apart from COG and CAZy functional analysis, we also performed a KEGG functional analysis of the collected fecal samples. Overall, the enriched functional profiles were divided into six categories, and the most highly enriched category was metabolism, followed by environmental information processing, genetic information processing, human diseases, cellular processes, and organismal systems (Figure 7a). In level two of KEGG analysis, carbohydrate metabolism was the most highly enriched category, followed by amino acid metabolism, energy metabolism, metabolism of cofactors and vitamins, glycan biosynthesis and metabolism, membrane transport, nucleotide metabolism, and lipid metabolism. These results suggest that the gut microbiota of the South China tiger possesses genetic potential related to metabolic functions, particularly in carbohydrate metabolism, which is crucial for host energy acquisition and nutrient utilization. Furthermore, the increased abundance of genes associated with environmental and genetic information processing suggests that these microbial communities play important roles in environmental adaptation and genetic variation (Figure 7b). As per the KEGG database enrichment analysis, Metabolic pathways, Biosynthesis of secondary metabolites, Microbial metabolism in diverse environments, Biosynthesis of cofactors, ABC transporters, Biosynthesis of amino acids, Two component system, Carbon metabolism, Ribosome, and Purine metabolism were the most highly enriched functional categories (Figure S3). The abundance differences in KEGG pathways between the LP and FM groups were compared based on the Wilcoxon ranksum test. Compared with the FM group, the LP group showed significantly higher abundances in energy metabolism, metabolism of other amino acids, drug resistance: antimicrobial, infectious disease: bacterial, and cell motility, whereas significantly lower abundances were detected in membrane transport, amino acid metabolism, glycan biosynthesis and metabolism, biosynthesis of other secondary metabolites, and endocrine system (Figure 7c).

### 3.4. The Composition Analysis of Antibiotic Resistance Genes

**Figure 8:**
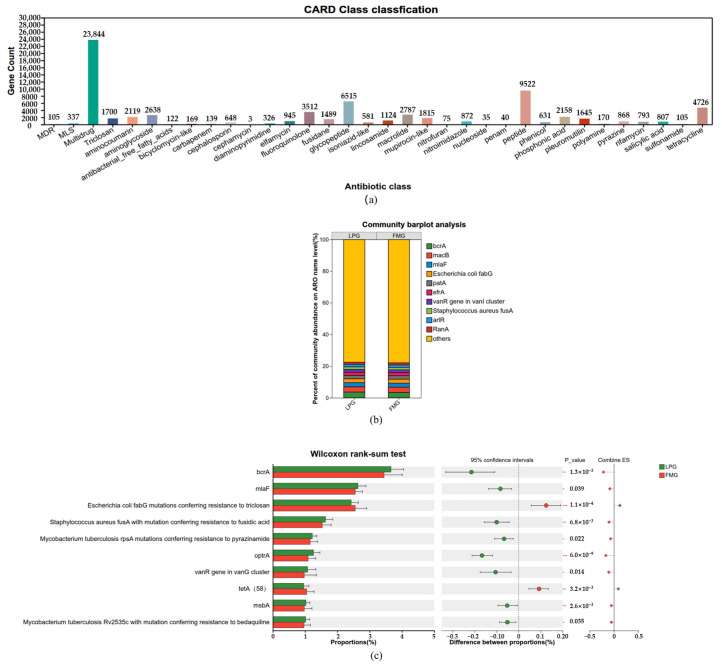
ARGs analysis in the gut microbiota of LP and FM groups. (**a**) ARG Counts per ARG Classes. (**b**) The relative abundance of ARGs. (**c**) Differential ARGs between LP and FM groups identified by the Wilcoxon ranksum test. Significance levels: * 0.01 < *p* ≤ 0.05, ** 0.001 < *p* ≤ 0.01, *** *p* ≤ 0.001.

A total of 1251 ARGs were identified across the collected fecal samples. Among these ARGs, several core resistance categories were detected, including resistance to multidrug, peptides, glycopeptides, tetracyclines, fluoroquinolones, and macrolides (Figure 8a). These resistance categories collectively constituted the core resistome of the gut microbiota in South China tigers.

The top 10 most abundant ARG categories showed nearly identical composition patterns between the LP and FM groups (Figure 8b). The abundance differences in ARG categories between the LP and FM groups were compared based on the Wilcoxon ranksum test. Compared with the FM group, the LP group exhibited significantly higher abundances of bcrA, mlaF, *Staphylococcus aureus* fusA with mutations conferring resistance to fusidic acid, *Mycobacterium tuberculosis* rpsA mutations conferring resistance to pyrazinamide, optrA, vanR (in the *vanG* cluster), msbA, and *Mycobacterium tuberculosis* Rv2535c mutations conferring resistance to bedaquiline, whereas significantly lower abundances were detected in *Escherichia coli* fabG mutations conferring resistance to triclosan, tetA(58) (Figure 8c).

## 4. Discussion

The South China tiger was listed as endangered on the International Union for Conservation of Nature’s Red List of Threatened Species. In recent years, numerous studies have investigated the composition and functional potential of the gut microbiota in tigers; however, studies focusing specifically on the gut microbiome of the South China tiger remain scarce. Live prey feeding more closely mimics natural hunting behaviour, and the nutritional condition experienced by tigers in the wild, and previous studies have demonstrated that feeding whole prey to felids confers benefits to gastrointestinal health [18,19]. In the current study, we investigated the composition and functional structure of the gut microbiota collected from the South China tiger fecal samples under different dietary treatments.

Previous studies have reported that gut microbial diversity is higher in captive Amur tigers than in their wild counterparts [11], and similar patterns have been observed in other mammals, including wolves [12], deer [13] and antarctic seal [20]. However, contrasting findings have also been reported, with some studies showing higher gut microbial diversity in wild tigers or no significant differences between wild and captive populations [1,21], highlighting the context-dependent nature of gut microbiome variation. In the present study, alpha diversity analyses revealed that the LP group exhibited higher overall microbial diversity and richness compared with the FM group, although no significant differences were detected for the Chao indices. Notably, increased microbial diversity does not necessarily indicate a healthier gut ecosystem, but may instead reflect ecological instability or increased exposure to external disturbances, particularly under captive conditions with substantial human intervention. Consistent with this interpretation, both PCoA and NMDS analyses demonstrated significant compositional differences between the two dietary groups. These findings indicate that, even under captive conditions, differences in food processing methods can substantially influence gut microbial diversity and community structure in the South China tiger. These differences may be associated with the requirement for storage and thawing processes in frozen meat, as well as individual differences of tigers in dietary adaptation. In contrast, live prey retains intact and fresh animal tissues, which may more effectively select for microbial communities adapted to the strict carnivorous physiology of tigers [22,23].

Consistent with previous studies on the South China tiger [1], Siberian tiger [11,24,25], Bengal tiger [26,27,28], and Malayan tiger [29], the gut bacterial community of the South China tiger in the present study was predominantly composed of *Bacillota*, *Actinomycetot*, *Pseudomonadota*, *Bacteroidota*, and *Fusobacteriota*. The consistent dominance of these bacterial phyla across different tiger subspecies suggests that they constitute a conserved core gut microbiota, playing essential roles in maintaining intestinal homeostasis and metabolic functions in tigers. Among them, *Bacillota* is known to be critically involved in nutrient absorption, fiber degradation, and immune modulation, and represents a dominant phylum in the gut microbiota of many mammalian species [30,31]. In the present study, differences in food processing methods did not alter the dominant status of *Bacillota* in the gut microbiome of the South China tiger. Although no statistically significant differences were observed (*p* > 0.05), the relative abundance of *Bacillota* showed a higher trend in the LP group compared with the FM group. At the genus level, differences in food processing methods did not alter the dominant bacterial genera in the gut microbiota of the South China tiger, which were mainly composed of *Clostridium*, *Collinsella, Blautia*, *Peptacetobacter*, *Escherichia*, *Lactococcus*, *Enterococcus*, *Mediterraneibacter*, and *Paeniclostridium*. This community structure is largely consistent with previous reports on the gut microbiota of the South China tiger [1,27]. Nevertheless, marked differences in the relative abundance of specific genera were observed between dietary groups. Several genera, including *Clostridium*, *Collinsella*, *Blautia*, *Fusobacterium*, and *Bacteroides*, have been reported to respond to high protein and high fat diets, particularly with increasing protein intake [32,33]. Although both groups in the present study received diets derived from the same animal sources, live prey feeding represents a more complex dietary environment than frozen meat, as it includes a broader range of animal tissues and associated microbial inputs. In this context, the relative abundance of Clostridium was significantly higher in the LP group than in the FM group, whereas no significant differences were observed for *Blautia*, *Fusobacterium*, or *Bacteroides*. This pattern suggests that the increase in Clostridium abundance may be associated with functions beyond energy acquisition alone. Notably, *Clostridium* comprises a taxonomically and functionally diverse group. Species level differential analysis revealed that several potentially pathogenic species, including *Clostridium perfringens*, *Clostridium tetani*, *Clostridium novyi*, *Clostridium botulinum*, and *Clostridium sordellii*, were significantly enriched in the LP group. At the same time, multiple beneficial or metabolically important species, such as *Clostridium butyricum*, *Clostridium fallax*, *Clostridium beijerinckii*, *Clostridium cellulovorans*, and *Clostridium aciditolerans*, also showed significant increases [34] (Table S4). Together, these findings indicate that live prey feeding does not unidirectionally promote either beneficial or pathogenic bacteria. Instead, by introducing more complex animal tissues and microbial sources, live prey feeding appears to enhance ecological niche complexity and functional diversity within the gut microbiota of the South China tiger.

In addition, live prey feeding resulted in a significant increase in the relative abundance of *Paraclostridium*, *Peptostreptococcus*, and *Peptoniphilus*. Previous studies have linked these genera to impaired intestinal barrier function, enhanced inflammatory responses, and dysregulated immune modulation, suggesting that their enrichment may reflect an elevated risk of intestinal inflammation or physiological stress [35,36,37]. However, the specific consequences of these microbial shifts for host physiology remain to be validated by integrating host immune parameters and histological evidence. Previous studies have also demonstrated that the abundance of *Slackia* in the gut microbiota is relatively stable, serving as an indicator of normal bile acid metabolism and protein fermentation capacity. In the present study, the relative abundance of *Slackia* was significantly reduced in the LP group compared with the FM group, which may be indicative of disrupted digestive function or the development of intestinal inflammation [38,39]. This suggests that although live prey feeding may promote convergence of the gut microbial community in South China tigers, it may simultaneously pose a potential risk.

Proteus is one of the main bacterial species responsible for digestive system diseases in tigers. In severe cases, they can even lead to death [40]. In the present study, the relative abundance of Proteus was significantly higher in the gut microbiota of the FM group compared with the LP group. This phenomenon may be attributed to the competitive balance established between the indigenous complex microflora in the tiger’s intestines and Proteus after the tigers ingested the internal organs of live prey, coupled with the strong adaptability of the host to microbial communities. However, in the present study, the frozen meat fed group created favorable conditions for the proliferation of Proteus due to factors including low temperature selection effects, carcass contamination of the intestines, resulting in reduced microbial competition and thereby conferring a proliferative advantage to Proteus [41,42].

Functional gene analysis represents a core component of gut microbiome research and provides critical insights into how microbial communities participate in and regulate host metabolic processes [16,17]. In this study, metagenomic data were systematically analyzed using the COG, CAZy, and KEGG functional databases. The results showed that differences in food processing methods did not alter the overall functional gene composition of the gut microbiota in the South China tiger; however, pronounced shifts in the relative abundance of multiple functional categories were observed. This indicates that dietary processing influences gut microbial metabolic characteristics primarily by modulating the distribution of functional potential rather than restructuring the fundamental functional framework. COG annotation revealed that a large proportion of microbial genes in the South China tiger gut were enriched in amino acid transport and metabolism, as well as carbohydrate transport and metabolism, reflecting a highly active metabolic profile that is consistent with the strict carnivorous diet of tigers. Differential analysis further demonstrated that, compared with the FM group, the LP group showed significant enrichment in functional categories related to cell cycle control, cell division, chromosome partitioning, cell motility, and cytoskeleton. In contrast, the FM group exhibited higher abundances of genes involved in carbohydrate transport and metabolism, defense mechanisms, and RNA processing and modification. This functional divergence suggests that the gut microbiota of the LP group is better adapted to a more complex dietary environment with higher exposure risks, adopting functional strategies characterized by enhanced microbial proliferation, motility, and environmental responsiveness. Conversely, the gut microbiota of the FM group appears to be optimized for a relatively stable and nutritionally simpler environment, relying on efficient substrate transport and metabolic processes to maximize energy utilization. Based on CAZy annotation, a total of 560 distinct CAZy enzymes were identified in the gut microbiota of the South China tiger, with GHs and GTs being the most abundant families. Studies have shown that GHs and GTs facilitate the efficient utilization of complex carbohydrate substrates derived from diet components and host-associated glycans, thereby contributing to overall host energy metabolism, which are important for nutrient acquisition from animal biomass in big cats [43]. Consistent with this observation, similar enrichment patterns of glycosyltransferase families have been reported in the gut microbiome of the Siberian tiger [24].

Meanwhile, metabolic pathway analysis using the KEGG dataset demonstrated enrichment of carbohydrate metabolism, followed by amino acid metabolism, energy metabolism, metabolism of cofactors and vitamins, glycan biosynthesis and metabolism, membrane transport, nucleotide metabolism, and lipid metabolism. These results indicate that the gut microbiome of the South China tiger exhibits high metabolic activity, particularly in carbohydrate-related pathways, which play a critical role in energy acquisition and nutrient utilization in carnivorous hosts. In addition, a notable enrichment of purine metabolism pathways was observed in the gut microbiota. Given that meat-based diets are rich in purines, the enrichment of microbial purine metabolism pathways may facilitate efficient degradation of uric acid, thereby preventing its excessive accumulation and associated health risks in carnivorous animals [44]. The enrichment of purine metabolism observed in this study is consistent with previous reports linking this pathway to purinerich diets in obligate carnivores and other high meat-consuming species [43]. Comparative analysis further revealed distinct functional adaptations of the gut microbiota between feeding regimes. In the LP group, pathways associated with metabolism of other amino acids, antimicrobial resistance, infectious diseases: bacterial, and cell motility were significantly upregulated. These functional shifts likely reflect a more complex dietary environment, in which the microbiota must adapt to diverse amino acid substrates while simultaneously coping with increased exposure to environmental and prey associated microorganisms, thereby enhancing microbial mobility and defensive capacities. In contrast, the FM group showed significantly higher enrichment in pathways related to membrane transport, amino acid metabolism, glycan biosynthesis and metabolism, biosynthesis of other secondary metabolites, and the endocrine system. This functional profile suggests that the gut microbiota of FM group individuals is more strongly oriented toward efficient nutrient transport, metabolic processing, and host microbe metabolic coordination, thereby maximizing energy utilization efficiency.

The emergence and dissemination of antibiotic-resistant bacteria (ARB) have become issues of increasing global concern. Substantial evidence has documented the widespread occurrence and transmission of ARB in diverse mammalian hosts [45,46]. In this study, we performed a comprehensive characterization of ARGs in the gut microbiota of captive South China tigers. A total of 1251 unique ARGs were identified, conferring resistance to multiple antibiotic classes, including peptides, glycopeptides, tetracyclines, fluoroquinolones, and macrolides. Notably, more than half of the detected ARGs (58.35%) were shared across individuals, indicating a high degree of ARG overlap within the gut microbial communities of captive tigers. This widespread sharing of ARGs is likely associated with the captive environment, which may facilitate the colonization and persistence of antibiotic-resistant bacteria through shared habitats, diets, and prolonged exposure to human-associated environments [47,48,49]. In addition, the similar microbial communities in frozen meat and live prey may also contribute to the observed overlap in ARG profiles between the two feeding groups. Further comparison of ARG profiles between the LP and FM groups revealed distinct distribution patterns associated with feeding regimes. In the LP group, several ARGs and resistance-associated mutations exhibited significantly higher abundances, including bcrA, mlaF, Staphylococcus aureus fusA mutations conferring resistance to fusidic acid, Mycobacterium tuberculosis rpsA mutations associated with pyrazinamide resistance, optrA, vanR within the vanG gene cluster, msbA, and Mycobacterium tuberculosis Rv2535c mutations conferring resistance to bedaquiline. Source tracking analysis indicated that these high abundance ARGs and resistance-conferring mutations were primarily derived from bacterial taxa such as Enterococcus, Staphylococcus, and Mycobacterium. The enrichment of these taxa associated ARGs in the LP group suggests that liv prey feeding introduces a more complex microbial reservoir, potentially increasing exposure to environmental and prey associated resistant bacteria and thereby reshaping the gut resistome [50,51,52]. These findings suggest that live prey feeding may increase the exposure of South China tigers to resistant bacteria from the environment and prey by introducing more complex microbial sources, particularly the microbial communities harbored in the gastrointestinal tracts of prey, thereby reshaping the profile of ARGs in their gut. In contrast, the frozen meat group showed significant enrichment of fabG and tetA(58). This result may be closely associated with the relatively simple nutritional composition of frozen meat, as well as the selective pressures generated during processing and storage. On the one hand, low temperature storage exerts inhibitory or lethal effects on certain temperature-sensitive microbes. On the other hand, exposure to disinfectants (such as triclosan) during frozen meat processing may impose directional selective pressure, thereby promoting the enrichment of resistant bacteria, including *Escherichia coli* strains with enhanced tolerance to both low temperatures and disinfectants [53,54]. Overall, although different feeding modes did not significantly alter the overall ARG composition in the gut microbiome of captive South China tigers, they markedly shaped the differential abundance profiles of ARGs by influencing the complexity of microbial sources, selective pressures associated with processing environments, and intermicrobial competitive interactions.

Despite these findings, several limitations should be acknowledged in this study. The relatively small sample size, potential variation in health status and age distribution, as well as the high level of relatedness resulting from historical inbreeding in the South China tiger population, may limit the strength of conclusions regarding their influence on gut microbiome composition. Given the critically endangered status of the South China tiger and the absence of extant wild populations, studies on this subspecies are inherently constrained by limited sample availability. Therefore, the present study primarily aimed to explore how dietary regimes that more closely resemble natural feeding conditions may be associated with variations in the gut microbiome of captive South China tigers. Accordingly, the findings from this study should be interpreted as preliminary and not generalized beyond the studied cohort. Future studies including larger sample sizes and additional institutions will be necessary to further validate these findings.

## 5. Conclusions

This study provides a preliminary characterization of the gut microbiome of captive South China tigers through metagenomic analysis. It systematically elucidates the gut microbial community structure, functional metabolic profiles, and ARGs, and clarifies the impact of food processing methods on the gut microbiota and resistome of captive South China tigers. Although the core structure and functional framework of the gut microbiome remain unchanged, food processing induces specific alterations in microbial abundance, metabolic pathways, and ARG profiles. These findings offer scientific support for optimizing precision feeding strategies for captive South China tigers, and contribute to the development of more effective species conservation strategies for endangered felids.

## Data Availability

The original contributions presented in this study are included in the article/Supplementary Material. Further inquiries can be directed to the corresponding author(s).

## Supplementary Materials

The following supporting information can be downloaded at: https://www.mdpi.com/article/10.3390/vetsci13030307/s1, Table S1: Metadata and sequencing characteristics of South China tiger fecal samples; Table S2: Functional category prediction based on genes extracted from South China tiger fecal sample base on the CAZy database; Table S3: Significant differences between the LP and FM groups based on the Wilcoxon ranksum test; Table S4: Significant differences between LP and FM groups at the Species Level.

## Abbreviations

LP	Live prey
FM	Frozen meat
CAZy	Carbohydrate-active enzymes
COG	Clusters of Orthologous Groups
ARGs	Antibiotic resistance genes
KEGG	Kyoto Encyclopedia of Genes and Genomes
